# Retail Packaging Affects Colour, Water Holding Capacity, Texture and Oxidation of Sheep Meat more than Breed and Finishing Feed

**DOI:** 10.3390/foods11020144

**Published:** 2022-01-06

**Authors:** Minh Ha, Robyn Dorothy Warner, Caitlin King, Sida Wu, Eric N. Ponnampalam

**Affiliations:** 1 School of Agriculture and Food, Faculty of Veterinary and Agricultural Sciences, The University of Melbourne, Parkville, VIC 3010, Australia; robyn.warner@unimelb.edu.au (R.D.W.); caitlink1@student.unimelb.edu.au (C.K.); sidaw1@student.unimelb.edu.au (S.W.); 2 Animal Production Sciences, Agriculture Victoria Research, Department of Jobs, Precincts and Regions, Bundoora, VIC 3083, Australia; Eric.Ponnampalam@agriculture.vic.gov.au

**Keywords:** Merino, composite, modified atmosphere packaging, trigas, camelina, lipid oxidation, colour stability, meat

## Abstract

This study investigated the CIELab colour, water holding capacity, texture and oxidative stability of sheep meat from different breeds, finishing feeds, and retail packaging methods. Leg primal cuts from a subset of Composite wether lambs (*n* = 21) and Merino wether yearlings (*n* = 21) finished on a standard diet containing grain and cereal hay, a standard diet with camelina forage, or a standard diet with camelina meal, were used in this study. *Semimembranosus* and *Vastus lateralis* were packaged in vacuum skin packaging (VSP), or modified atmosphere packaging with 80% O_2_ and 20% CO_2_ (HioxMAP), or with 50% O_2_, 30% N_2_, and 20% CO_2_ (TrigasMAP). Packaging had a greater effect (*p* < 0.001) on L*, a*, b*, hue, and chroma than the effects from breed and finishing feed. Purge loss was affected by packaging. Cooking loss was affected by breed for *Semimembranosus* and packaging for both muscle types. HioxMAP and TrigasMAP increased WBSF and Texture Profile Analysis hardness of the meat compared to VSP. Lipid oxidation, assessed by TBARS, were lower in camelina forage or camelina meal supplemented diets and TrigasMAP compared to standard diet and HioxMAP, respectively. Total carbonyl and free thiol content were lower in VSP. Thus, supplementing feed with camelina forage or meal and lowering oxygen content in retail packaging by TrigasMAP or VSP are recommended to ensure optimal sheep meat quality.

## 1. Introduction

In the sheep meat supply chain, quality traits such as colour, water holding capacity, texture, and oxidative stability are determined by various factors, including breed, finishing feed, and retail packaging method. High-oxygen modified atmosphere packaging (HioxMAP), using 70−80% O_2_ and 20−30% CO_2_, is a common meat retail packaging method due to its ability to maintain the “fresh” cherry red colour of meat. However, extensive research has shown an increased oxidation and lower eating quality of meat in HioxMAP compared with vacuum skin packaging (VSP) [1,2,3]. Trigas modified atmosphere packaging (TrigasMAP) is a more recently developed method in which oxygen is partially substituted with an inert gas, e.g., nitrogen, and has shown promising results in improving the eating quality and shelf life of meat [4].

The finishing feed of livestock is another factor affecting the quality of meat through altering the antioxidant activity in post-mortem muscles. The incorporation of antioxidants, such as vitamin E, or antioxidant-rich pasture crops were shown to result in reduced lipid oxidation and improved eating quality [5,6]. The use of oil crops and meals as animal feed supplements from the Brassica family, particularly camelina (Camelina sativa) has recently gained attention in improving animal productivity and carcass value [7]. Camelina seed is known to contain essential fatty acids, such as alpha-linolenic acid and different phenolic compounds such as flavonoids and proanthocyanidins, which provides an opportunity to improve the oxidative stability of meat [8].

Animal breed or the genetic background is also known to influence the sheep meat quality. For example, pure Merino sheep is believed to produce meat that is less tender or darker in colour compared with meat from crossbred sheep. The differences in texture and colour are associated with carcass fatness, muscle glycogen concentration, muscle iron concentration, and/or post-mortem chill effects [9,10].

Many studies have demonstrated the effect of the three supply chain factors of breed, feed and packaging individually. However, little is known about their interactive effect or the extent to which each factor affects meat quality. Thus, the aim of this study was to investigate the quality of sheep topsides (*Semimembranosus*) and knuckles (*Vastus lateralis*) from Merino yearlings and Composite wether lambs, finished on three different diets (standard diet; standard diet supplemented with camelina forage; or standard diet supplemented with camelina meal), and packed in three retail packaging methods (VSP, HioxMAP or TrigasMAP). The CIELab colour, water holding capacity, texture, lipid oxidation and protein oxidation were measured.

## 2. Materials and Methods

### 2.1. Animal Housing and Feeding

Feeding experiments were conducted at the Agriculture Victoria Research, Hamilton Centre, Hamilton, VIC, Australia. All animal procedures were conducted in accordance with the Australian code for the care and use of animals for scientific purposes (National Health and Medical Research Council 2013). Animal ethics approval was granted by the Department of Jobs, Precincts and Regions (DJPR) Agricultural Research and Extension Animal Ethics Committee (AEC Code No: 2016-17). Details of the experimental design, feeding of animals, and slaughter procedures were described previously [7]. In brief, a subset of maternal Composite wether lambs (*n* = 21) and Merino wether yearlings (*n* = 21) kept in different pens selected based on their final liveweights were used for this study. The primal cuts were from animals randomly allocated to three finishing feeds: a standard pelleted diet containing grain and cereal hay (SPD), a pelleted mixture diet containing 15% camelina forage hay (SCF), or a pelleted mixture diet containing 8% camelina meal (SCM). Diets were formulated using the ingredients available in the major sheep producing regions. The metabolizable energy (ME) and crude protein concentrations of the diets were managed to be 10−11 MJ ME/kg dry matter and 14−15% crude protein.

### 2.2. Slaughter Procedure and Collection of Sheep Primals

The animals were transported approximately 250 km using a semi-trailer to a commercial abattoir and slaughtered after 18 h in lairage. At 5 days post-mortem, legs from the left side of the animals were collected. Topsides and knuckles from the legs were boned from the legs. The muscles were vacuum-packed using a Multivac C450 (Sepp Haggenmüller GmbH & Co., Wolferschwenden, Germany) with Cryovac^®^ vacuum pouches (PA/PE 70, Sealed Air, Fawkner, VIC, Australia) with an oxygen permeability less than 65 cc/m^2^/24 h and water transmission less than 5 g/m^2^/24 h. The vacuum-packed muscles were frozen at −20 °C for 6 weeks.

### 2.3. pH, Cutup, Packaging and Retail Display

Following thawing at 2 °C for 24 h, meat cutup and packaging were conducted at approximately 6 °C. Prior to cutup, the pH of the muscle was measured using a spear-head pH probe attached to a WP-80 pH-mV-temperature meter (TPS Pty Ltd., Brisbane, QLD, Australia). *Semimembranosus* and *Vastus lateralis* were extracted from the topsides and knuckles. The muscles were cut into three sections from the anterior and randomly allocated to VSP, HioxMAP, or TrigasMAP packaging treatments. All packaging was conducted using a Multivac T200 (Sepp Haggenmüller GmbH & Co., Wolferschwenden, Germany). Meat samples were placed on a cello soaker pad (130 mm × 90 mm; CBS, Carrum Downs, Australia) inside a black Cryovac^®^ MAP packaging tray (T0D0901C 170 mm × 223 mm, Sealed Air, VIC, Australia). The trays were sealed with a Biaxially Oriented PolyAmide/Polyethylene/Ethylene vinyl alcohol-based film (OTR 15 cc/m^2^/24 h). The gas composition in HioxMAP was 80% O_2_ and 20% CO_2_ while TrigasMAP had 50% O_2_, 30% N_2_, and 20% CO_2_. Vacuum skin packaging was conducted using Cryovac^®^ Darfresh^®^ film (OTR 4 cc/m^2^/24 h) and black Cryovac^®^ trays (Sealed Air, Fawkner, VIC, Australia). Packaged samples were stored in a simulated retail display cabinet with LED lighting (~310 lux, Bromic Refrigeration, Ingleburn, NSW, Australia) at 4 °C for 10 days. The samples were rotated daily between shelves to minimise the effects of variations in illumination and temperature within the retail display cabinet on the samples.

### 2.4. Instrumental Colour Measurement

After 10 days in simulated retail display, CIE L* (lightness), a* (redness), and b* (yellowness) were measured on the meat surface using a Minolta chroma meter CR-300 (Minolta Co., Ltd., Osaka City, Japan), calibrated with a white plate (no. 20733120; Y = 84.9; x = 0.3171; y = 0.3240). The colour of vacuum skin-packed samples was measured after a 30 min blooming at 6 °C, whereas the colour of HioxMAP and TrigasMAP samples were measured immediately after the samples were removed from packaging. Duplicate colour measurements were taken on each sample. Hue angle (h*) and chroma (C*) were calculated using the following equations:

Hue angle = arctan (b*/a*)

$$\mathrm{Chroma}=\sqrt{{\left(a^{*}\right)}^{2}+{\left(b^{*}\right)}^{2}}\;$$

### 2.5. Purge and Cooking Loss

Purge loss was expressed as the weight loss in packaging during retail display. Samples were weighed before packaging (initial weight) and after 10 days storage (final weight). Excess moisture was removed with paper towel before weighing. Purge loss was calculated using the following equation:

Purge loss (%) = (weight before pack − weight after pack)/(weight before pack) × 100

Cooking loss was measured during the cooking procedure for Warner-Bratzler shear force and texture profile analysis. Each sample was weighed before cooking. After cooking, excess moisture on the meat surface was removed with paper towel before weighing. Cooking loss was calculated as:

Cooking loss (%) = (weight before cook − weight after cook)/(weight before cook) × 100

### 2.6. Warner-Bratzler Shear Force

Warner-Bratzler shear force were measured according to Peng et al. [11]. Samples were individually placed in a plastic bag in temperature-equilibrated water baths (F38-ME, Julabo, Seelbach, Germany) set at 75 °C and cooked to internal temperature of 71 ± 0.5 °C. The internal temperature was monitored using T-type thermocouples inserted to the middle of meat samples and the thermocouples were connected to a Grant Squirrel Series 2020 datalogger (Grant Instruments Ltd., Cambridge, UK). After cooking, the samples were chilled in an ice water bath for 30 min and stored at 4 °C overnight. Six 4 cm long rectangular strips with 1 cm × 1 cm cross section area were obtained from each sample by cutting parallel to the muscle fibres. Each strip was sheared using a Lloyd Instruments LRX Materials Testing Machine (Lloyd Instruments Ltd., Hampshire, UK) equipped with a 5000 N load cell and a V-shape Warner-Bratzler shear force blade at an extension rate of 300 mm/min. The WBSF (N) of each sample was expressed as the average peak force of measurements from the six strips.

### 2.7. Texture Profile Analysis (TPA)

Texture profile analysis was measured using a double bite compression procedure outlined previously Peng et al. [11]. A piece of meat measuring 1 cm in thickness was obtained from each sample. The meat was compressed twice at the same position by a 6.3-mm diameter plunger which was driven 8 mm into the sample at a crosshead speed of 50 mm/min using Lloyd Instruments LRX Materials Testing Machine (Lloyd Instruments Ltd., Hampshire, UK) equipped with a 5000 N load cell. Hardness (N) (first bite compression), cohesiveness, and chewiness (N) were measured. TPA values for each sample were averaged from six measurements.

### 2.8. Lipid Oxidation

Lipid oxidation in meat was assessed by 2-thiobarbituric reactive substances (TBARS) procedure as reported by Sorensen and Jorgensen [12] with modifications. For each sample, duplicate (5 g) from each sample were finely chopped and homogenised in 12.5 mL of chilled (4 °C) trichloroacetic acid (TCA) solution (20% TCA (*w*/*v*) in 2 M phosphoric acid) at 12,000 rpm for 1.5 min using a Polytron PT 10–35 GT homogeniser (Thermo Fisher Scientific, VIC, Australia). The homogenate was then centrifuged at 1800× *g* using a Rotina 380R Hettich Centrifuge (LabGear, South Melbourne, VIC, Australia) for 10 min at 4 °C. The supernatant was filtered using Whatman filter paper no. 1. Equal volumes of the filtrate and 5 μM 2-thiobarbituric acid (TBA) were mixed and incubated in a test tube at 95 °C for 60 min. Following incubation, the tube was placed on ice for 15 min. Absorbance at 532 nm was measured for duplicate aliquots from each tube using a Multiskan spectrophotometer (Thermo Fisher Scientific, Scoresby, VIC, Australia). Malondialdehyde (MDA) was quantified against a standard calibration curve with 1,1,3,3-tetraethoxypropane (TEP). Results were expressed as mg MDA·kg^−1^ meat.

### 2.9. Total Carbonyl Content

Carbonyl content of the meat samples was determined according to Lund et al. [13] with modifications. Briefly, 1 g of meat samples were homogenised for 1 min at 15,000 rpm in 15 mL of homogenisation buffer (2.0 mM Na_4_P_2_O_7_, 10 mM Tris-maleate, 2 mM EGTA, 100 mM KCl, pH 7.4) using a Polytron PT 10–35GT homogeniser (Thermo Fisher Scientific, VIC, Australia). Two equal aliquots (0.5 mL) from the homogenate were washed with a HCl:acetone (3:100 *v*/*v*) solution three times followed by washing with 10% (*w*/*v*) TCA twice. Out of the two identical samples, (i) 0.5 mL of DNPH dissolved in 2 M HCl was added to the first sample for carbonyl derivatisation and (ii) 0.5 mL of 2 M HCl was added to the other sample for protein concentration determination. Absorbance of the samples were measured at 280 nm to determine protein concentration against a standard curve with BSA (Sigma-Aldrich, Castle Hill, NSW, Australia); and, at 370 nm to determine total carbonyl content. Carbonyl concentration was determined by using the absorption coefficient at 370 nm for the hydrazones formed (22,000 M^−1^·cm^−1^) against the protein concentration and expressed as nmol·mg^−1^ protein.

### 2.10. Free Thiol Content

To determine the loss of thiol (sulfhydryl) groups, the 5,5′-Dithiobis (2-nitrobenzoic acid) (DTNB) method was used as described by Lund et al. [13]. Duplicates (2 g) of each sample were homogenised at 16,000 rpm in 40 mL of 5% (*w*/*v*) sodium dodecyl sulfate (SDS) in 0.1 M Tris buffer (pH 8) using a Polytron PT 10–35 GT homogeniser (Thermo Fisher Scientific, Scoresby, VIC, Australia). The homogenates were incubated at 95 °C for one hour in covered test tubes. The samples were cooled and centrifuges for 20 min at 1200× *g* using a Rotina 380R Hettich centrifuge (LabGear, VIC, Australia). The supernatants were filtered using Whatman filter paper no 1 and the protein concentration of filtrates was determined at 280 nm using a standard curve with bovine serum albumin (BSA) (Sigma-Aldrich, Castle Hill, NSW, Australia). The samples were diluted to a protein concentration of 1.5 mg.mL^−1^ using the SDS homogenisation buffer. The diluted samples were used to determine thiol group concentration by adding 2 mL of 0.1 M Tris buffer (pH 8) and 0.5 mL DTNB to 0.5 mL of sample. Samples were incubated for 30 min in the dark and absorbance at 412 nm was measured using a Multiskan spectrophotometer (Thermo Fisher Scientific, VIC, Australia). The concentration of thiol groups was analysed against a standard curve of L-cysteine prepared in 5% (*w*/*v*) SDS in 0.1 M Tris buffer (pH 8). Total thiol content was calculated and expressed as nmol·mg^−1^ protein.

### 2.11. Statistical Analysis

Data were analysed using restricted maximum likelihood (REML) with GenStat 16th Edition (VSN International, Hemel Hempstead, UK). For pH before packaging, breed (Composite and Merino), feed type (SPD, SCF and SCM), and muscles (*Semimembranosus* and *Vastus lateralis*) were fitted as fixed effects. Pen and carcass number (all nested within, i.e., pen/carcass number) were fitted as random effects. For all other quality traits, breed (Composite and Merino), feed type (SPD, SCF and SCM), packaging method (HioxMAP, TrigasMAP and VSP) were fitted as fixed effects. Pen and carcass number (all nested within) were fitted as random effects. Separate analyses were conducted for each muscle type (*Semimembranosus* and *Vastus lateralis*). *p* < 0.05 was used as the level for significant differences.

## 3. Results

### 3.1. pH and Colour

**Figure 1:**
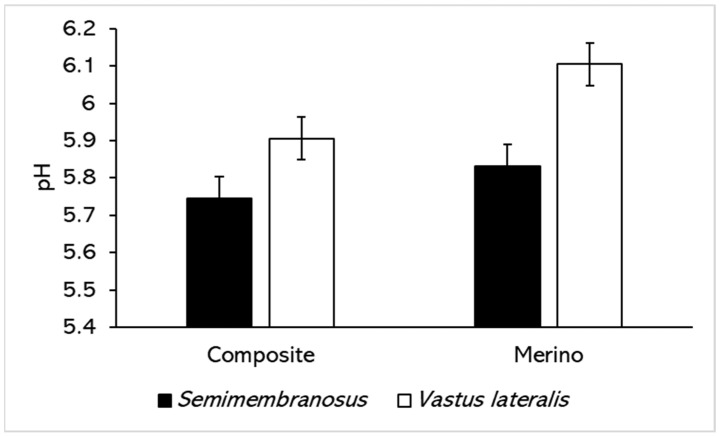
The pH of *Semimembranosus* and *Vastus lateralis* from Composite and Merino sheep before retail packaging. Values are predicted means ± standard error of differences (SED). *p* (breed × muscle) is 0.096.

Figure 1 shows that the pH of meat prior to packaging significantly differed between the Composite and Merino sheep (*p* = 0.004) and between the *Semimembranosus* and *Vastus lateralis* muscles (*p* < 0.001). While the pH of *Vastus lateralis* was higher compared to *Semimembranosus* for both breeds, the difference was more substantial in meat from Merino compared to Composite sheep. There was no significant effect of feed (*p* = 0.7) on the pH.

**Figure 2:**
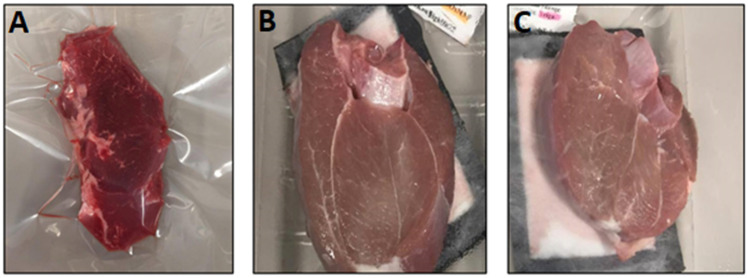
Visual comparison of *Vastus lateralis* from a Composite sheep finished on standard pelleted diet with grain and hay and packed in (**A**) vacuum skin packaging; (**B**) HioxMAP; or (**C**) TrigasMAP for 10 days at 4 °C.

Using the CIE L*, a*, and b*-values, the colour stability of lamb was evaluated (Table 1). Breed had differential effects on the lightness (L*) of *Semimembranosus* and *Vastus lateralis* muscles. Compared to Composite, Merino had a lower L* value for *Semimembranosus*, yet a higher L* value for *Vastus lateralis*. A significant effect of breed on a*, b*, and hue were also observed for *Vastus lateralis*, but not *Semimembranosus*. The finishing feed had no effect on any of the colour parameters. The packaging method had a greater influence on all colour parameters (*p* < 0.001 for all) compared to breed and feed effects. While there were small differences between HioxMAP and TrigasMAP, most colour parameters significantly differed between VSP and HioxMAP or between VSP and TrigasMAP for both muscles. Interestingly, when comparing VSP and HioxMAP, hue differed in *Semimembranosus*, but not *Vastus lateralis*. Together, these results show that the choice of packaging methods had a greater influence on the colour stability of sheep meat, compared to breed and feed, and the extent to which of HioxMAP negatively impacts meat colour was muscle dependent. A visual illustration of *Vastus lateralis* in the three packaging methods is presented in Figure 2.

### 3.2. Water Holding Capacity

**Figure 3:**
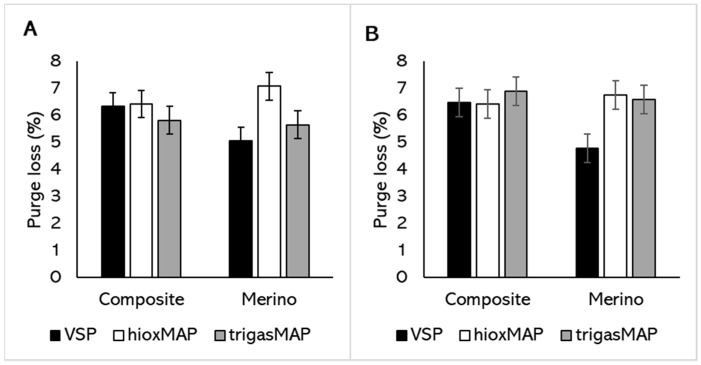
Purge loss of (**A**) *Semimembranosus* and (**B**) *Vastus lateralis* from two sheep breeds (Composite or Merino) in three retail packaging methods (VSP = vacuum skin packaging; HioxMAP = high-oxygen modified atmosphere packaging with 80% O_2_ and 20% CO_2_; or TrigasMAP = trigas modified atmosphere packaging with 50% O_2_, 30% N_2_ and 20% CO_2_). Values are predicted means ± standard error of differences (SED). *p* (breed × packaging method) values are 0.014 for *Semimembranosus* (**A**) and 0.015 for *Vastus lateralis* (**B**).

Water holding capacity was measured as purge and cooking losses (Table 2). While the three supply chain factors (breed, feed, and packaging method) appear to influence purge loss to a similar extent, only packaging method had a significant effect (*p* < 0.001) on purge loss. It is worth noting that purge loss of *Semimembranosus* in TrigasMAP (5.7% ± 0.3 SED) was similar to VSP (5.7% ± 0.3 SED) and lower than HioxMAP (6.8% ± 0.3 SED). There was no difference in purge loss of *Vastus lateralis* in HioxMAP and TrigasMAP, indicating differences between the two muscles in their response to water holding capacity. A significant interaction between breed and packaging method was also observed for purge loss (Figure 3). While the purge loss did not differ across the three packaging methods for composite sheep *Semimembranosus* and *Vastus lateralis*, TrigasMAP reduced the purge loss in Merino *Semimembranosus* compared to HioxMAP (Figure 3A). This reduction in purge loss by TrigasMAP was not observed for *Vastus lateralis* (Figure 3B). Packaging method had a significant effect on purge loss of *Semimembranosus* and *Vastus lateralis* from Merino, but not those from Composite sheep.

Merino *Semimembranosus* had a lower cooking loss compared to the same muscle type from Composite sheep (Table 2). Finishing feed did not affect cooking loss for either *Semimembranosus* or *Vastus lateralis*. The difference in cooking loss between the three packaging methods were significant with the lowest cooking loss in TrigasMAP (30.9% ± 0.5 SED), followed by HioxMAP (32.2% ± 0.5 SED) and VSP (35.1% ± 0.5 SED). No significant interactions were observed for cooking loss.

### 3.3. WBSF and Texture Profile Analysis

Breed or finishing feed had no effect on WBSF for either of the two muscles (Table 2). Differences in WBSF between the three packaging methods were only found for *Semimembranosus*, which were tougher in HioxMAP and TrigasMAP, compared to VSP. No significant interactions were observed for WBSF in either muscle types.

The effect of the three supply chain factors on Texture Profile Analysis hardness differed between the two muscles (Table 2). Within the *Semimembranosus* samples, hardness was affected by breed only, with *Semimembranosus* from Merino having a higher hardness value compared to *Semimembranosus* from Composite sheep. The hardness of *Vastus lateralis* was only affected by finishing feed, with SCM having a lower hardness value than that of SPD and SCF. Cohesiveness and chewiness were affected by breed and packaging method in both muscle types. Cohesiveness and chewiness were lower in VSP compared to HioxMAP and TrigasMAP for both muscle type, suggesting a softer texture in a low oxygen packaging environment. No significant interactions were observed for hardness, cohesiveness, and chewiness in either muscle types.

### 3.4. Lipid Oxidation

**Figure 4:**
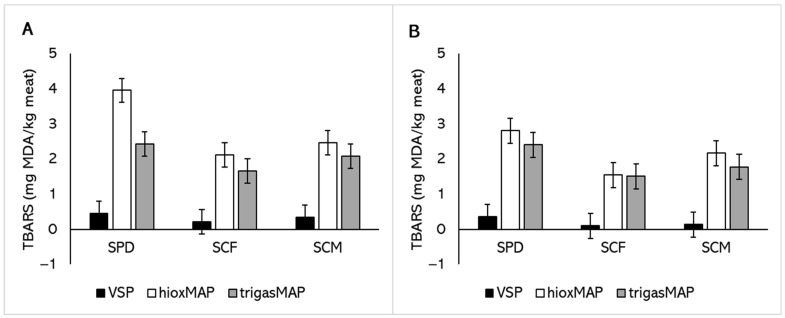
Thiobarbituric acid reactive substances (TBARS) values of (**A**) *Semimembranosus* and (**B**) *Vastus lateralis* from sheep finished on three diets (SPD = standard pelleted diet containing grain and cereal hay; SCF = pelleted mixture diet containing 15% camelina forage hay; or SCM = pelleted mixture diet containing 8% camelina meal) and retail displayed in three packaging methods (VSP = vacuum skin packaging; HioxMAP = high-oxygen modified atmosphere packaging with 80% O_2_ and 20% CO_2_; or TrigasMAP = trigas modified atmosphere packaging with 50% O_2_, 30% N_2_ and 20% CO_2_). Values are predicted means ± standard error of differences (SED). *p* (feed × packaging method) values are 0.011 for *Semimembranosus* (**A**) and 0.243 for *Vastus lateralis* (**B**).

Lipid oxidation in meat was assessed using TBARS assay and the levels were expressed as mg MDA/kg of meat. Breed did not affect lipid oxidation in either of the two muscle types (Table 3). However, supplementation of feed with either camelina forage or camelina meal led to a reduction in TBARS values compared to the standard pelleted diet containing cereal hay and grains. Packaging type not only had a significant effect but also to a greater extent (substantially higher coefficients) than feed on TBARS values. TrigasMAP was able to reduce lipid oxidation compared to HioxMAP for *Semimembranosus*, but not *Vastus lateralis.* There was also a significant interaction between finishing feed and packaging method for the *Semimembranosus* samples. Figure 4 shows that the TBARS value of meat in HioxMAP were substantially greater in the control diet (SPD) compared to the two camelina supplemented diets (SCF and SCM), especially for *Semimembranosus*. These results further emphasise the need for sheep meat to be packaged in a lower oxygen environment when sheep feed is not supplemented with antioxidants.

### 3.5. Protein Oxidation

There were no differences between breed and finishing feed treatments on total carbonyl and free thiol content in either *Semimembranosus* or *Vastus lateralis* (Table 3). On the other hand, protein oxidation significantly differed between the three packaging methods in both muscle types. Total carbonyl was significantly lower in VSP compared to HioxMAP and TrigasMAP. There was a small but significant difference between total carbonyl of HioxMAP and TrigasMAP with TrigasMAP inducing a lower carbonyl generation. The free thiol content values were significantly lower in VSP compared to either HioxMAP or TrigasMAP for both muscle types, suggesting that minimising protein oxidation in sheep meat can be achieved by the use of oxygen at a level below 50%. Within SPD treatment, Merino *Semimembranosus* or *Vastus lateralis* had lower free thiol contents compared to equivalent muscles from Composite sheep, indicating the importance of cameline in sheep finishing diets for Merino sheep to reduce protein oxidation. This difference was not observed for total carbonyl.

## 4. Discussion

### 4.1. Colour and pH

Differences in Instrumental CIELab parameters due to breed was more apparent in *Vastus lateralis* compared to *Semimembranosus*. Merino *Vastus lateralis* had higher L*, and lower a*, b*, hue, and chroma than the same muscle from Composite sheep. These results suggest that the meat from Merino sheep was less colour stable, which coincided with a substantially higher pH compared to meat from Composite sheep. This agrees with a previous study which found the fastest drop in oxy-/met-myoglobin ratio in meat from Merino, compared to other crossbreeds [14]. The lower colour stability in Merino sheep meat was also reported in other studies [9,10]. Furthermore, significant differences in fatty acid composition and vitamin E concentration were found in the *Longissimus* of Merino and crossbred sheep [7,10]. These differences are likely to result in variation in the oxidation of myoglobin, thus affecting the colour stability of meat.

Breed and pH have been shown to be among the most important predictors in sheep meat colour stability. Meat from Merino often has a different ultimate pH to meat from crossbreed sheep [10,14]. A study on lamb from the Australian Cooperative Research Centre for Sheep Industry Innovation showed that Merino *Longissimus* with a higher pH had the least colour stability in overwrap [14]. The link between breed, ultimate pH, and colour stability is complex. Meat pH has been linked to myoglobin autooxidation, changes in enzymatic activities, iron molecule oxidation, and light scattering, all of which affect the appearance of the meat [15]. It is worth noting that most studies on sheep meat have focused mainly on the *Longissimus*, which is known to differ from *Semimembranosus* and *Vastus lateralis* in muscle fibre type, contributing to differences in colour [15].

Myoglobin oxidation and oxygenation status is affected by the level of oxygen during retail packaging. This study shows that the storage of lamb under high (80%) and moderate (50%) oxygen environments for 10 days significantly reduces the colour stability of both *Semimembranosus* and *Vastus lateralis*, when compared to lamb stored in VSP. The significant decrease in L*, a*, b*, hue and chroma in HioxMAP, compared to VSP, are consistent with the results of previous studies [3,16,17]. Lower chroma and higher hue values are undesirable in red meat as it represents paler and duller meat [18]. The colour results from TrigasMAP in the present study suggest that after 10 days of storage, TrigasMAP does not offer enhanced colour stability, similar to results of Resconi et al. [16] in which beef were displayed in different O_2_ levels for up to 8 days. However, it is possible that meat in TrigasMAP with a shorter retail display time may have better colour than in HioxMAP. Zakrys et al. [19] suggested that 50% O_2_; 30% N_2_; 20% CO_2_ may provide opportunity for improved shelf life by enhancing the a* value of beef, compared to HioxMAP after a 3-day storage. Meat surface colour has been shown to deteriorate after three days of storage in HioxMAP [20]. This was attributed to the reduction of metmyoglobin reducing activity during prolonged storage, thereby favouring the oxidative process of oxymyoglobin to metmyoglobin. The study of Khliji et al. [21] indicated that consumers discriminate against red meat with a* values below 14.5. The a* values for both muscles in HioxMAP and TrigasMAP were well below this threshold for both muscles in this study. Thus, retail displaying of sheep meat in HioxMAP or TrigasMAP for 10 days is not recommended for colour enhancement.

### 4.2. Water Holding Capacity and Texture

Contradictory results have been reported for the purge loss of meat in different packaging treatments, while others reported an increase in the purge of meat under vacuum [22]. Taylor et al. [17] showed that the weight loss of vacuum-packed beef and pork was less than MAP (75%O_2_/25%CO_2_)-packed samples after storage. Similar results were reported in other studies [23,24]. However, it was suggested in a review by McMillin [25] that the purge loss of meat displayed under vacuum packaging is higher than MAP, partly attributed to the negative pressure. In the current study, the two muscles responded differently to the effect of packaging. HioxMAP led to a higher purge loss for *Semimembranosus* but lower purge loss for *Vastus lateralis* compared to VSP, emphasising that future purge loss investigations should consider muscle differences.

Unlike purge loss, similar cooking losses were observed for both muscle types with a higher cooking loss found for both HioxMAP and TrigasMAP compared to VSP, in agreement with previous studies [26]. On the other hand, cooking loss due to breed differences appear to be muscle-specific, with *Semimembranosus* from Merino having a lower cooking loss compared to the same muscle from Composite. While the underlying mechanisms of water holding capacity remains an ongoing research area, previous studies showed that variations in muscle fibre type and connective tissue composition play a role in cooking loss differences [27,28,29].

Packaging appeared to affect the two muscles differently. WBSF of *Semimembranosus* in HioxMAP and TrigasMAP were higher than VSP. Similar results have been reported for beef topside and beef round, where the beef topside is a more likely response to ageing than beef round muscle after storage [30]. Numerous studies have shown the negative effect of HioxMAP on sheep meat eating quality. Frank et al. [1] showed a significantly lower sensory tenderness of lamb in HioxMAP compared to VSP. Similar results on various texture measurements were also found for meat from other species [11,13,31]. Previous studies found while the WBSF of beef *Longissimus* did not differ between oxygen levels from 40–80%, sensory panellists preferred beef in lower O_2_ environments 40–50% [19,32,33]. Furthermore, various studies have established that the toughening of meat in HioxMAP is caused by increased protein oxidation resulting in more disulfide bond formation between actomyosin complexes, less degradation of structural proteins, e.g., desmin and troponin T, and deactivation of calpain [13,32,34,35,36]. It is worth noting that the exact mechanisms appeared to be muscle- and species-specific [2,3,34]. Our results on texture are consistent with the protein oxidation results, which showed that significant differences were only found when VSP was compared to HioxMAP and TrigasMAP. Together, these results indicate VSP is the preferred packaging method for lamb regardless of breed and finishing feed treatments.

### 4.3. Lipid Oxidation

Lipid oxidation is a key quality determinant in meat, as it causes the development of off-flavours and rancidity in meat. Free radical formation from lipid oxidation has also been linked to increased myoglobin oxidation and thus discolouration [37]. Feeding strategy of livestock can play a significant role in manipulating lipid oxidation of meat. The present study found a reduction of lipid oxidation of *Semimembranosus* and *Vastus lateralis* from sheep finished on diets supplemented with camelina forge or camelina meal. These findings compliment previous studies which found significant decreases in TBARS for forage fed animals when compared to grain-fed animals [5,38,39]. Lamb muscles finished on diets supplemented with camelina cake has been shown to have a different fatty acid composition compared to those on the standard pelleted diet without camelina supplementation [40]. Furthermore, we have reported in a separate study that both camelina hay- and camelia meal-supplemented diets reduced (*p* < 0.001) arachidonic acid concentration of *Longissimus* from these animals compared with the SPD diet [7]. In addition, the SCM diet significantly increased alpha linolenic acid (ALA) concentration of the *Longissimus* compared to SPD and SCF, resulting in an increase in total omega-3 concentration and the decrease in the ratio of *n* − 6/*n* − 3 in meat [7].

The packaging results in this study show that TrigasMAP is an effective method to reduce lipid oxidation in packaging, regardless of breed and finishing feed treatments, consistent with previous studies on the effect of varying oxygen content on lipid oxidation [19,34,41]. Reducing oxygen content in retail packaging is even more important when sheep is not finished on supplemented diet.

Consumers discriminate against the off-flavour of beef when TBARS reaches the 2.28 mg MDA/kg meat threshold [42]. While similar investigations are needed for sheep meat, the present findings suggest that retail display of sheep meat in HioxMAP for 10 days leads to unacceptable flavour regardless of breed, feed or muscles. Supplementation of finishing feeds with camelina forage and TrigasMAP offers the potential to reduce TBARS values to below this threshold, thus reducing the economic loss for the industry. It should be noted that VSP provided consistently minimal lipid oxidation regardless of breed, feed or muscle treatments.

### 4.4. Protein Oxidation

Protein oxidation during retail display has been shown to lead to changes in protein aggregation and degradation, with implication for meat tenderisation. Carbonyl content substantially increased after 10 days of retail display in both TrigasMAP and HioxMAP (Table 3). Interestingly, TrigasMAP reduced the extent of formation of carbonyl groups compared to HioxMAP. This is similar to that observed in lipid oxidation, and agrees with previous studies [19] which reported increases in carbonyl content with increases in oxygen concentration. This would suggest that reducing the oxygen concentration in the packaging system to 50% reduced the extent of post-mortem oxidative processes.

Morzel et al. [43], using an ·OH radical generating system from pig *Longissimus*, showed oxidation induced formation of disulfide bridge and protein polymerisation led to a reduction in proteolysis susceptibility of myofibril proteins. Free thiol groups (sulfhydryl) are susceptible to oxidation; therefore, the quantification is a useful measure to determine the extent of protein oxidation in muscle foods. The present study showed the free thiol content of both *Semimembranosus* and *Vastus lateralis* did not differ between breed and finishing feed treatments. However, significant differences were observed between VSP and HioxMAP and TrigasMAP treatments. Bao and Ertbjerg [34] reported no difference in free thiol content between 80% O_2_ and 60% O_2_ in HioxMAP packaged beef. The underlying mechanisms behind differences in free thiol content between Composite and Merino on SPD is not understood. However, differences in muscle fibre type, lipid content and composition, and antioxidant capacities between breeds are likely to be involved [44].

## 5. Conclusions

By examining the colour, water holding capacity, texture, and oxidative stability of sheep meat from different breeds, finishing feed, and retail packaging methods, this study demonstrated the complexity in how different sheep breeds and muscles respond to variations in finishing feeds and packaging methods. Packaging of sheep meat in low, moderate, or high oxygen environments affected the colour to a greater extent than breed and finishing feeds. However, supplementation of the finishing feed with either camelina forage or camelina meal significantly reduced the lipid oxidation of sheep meat. Understanding how and to which extent supply chain factors affect the quality of sheep meat enables sheep producers and processors to prioritise intervention strategies to ensure optimal quality.

## Figures and Tables

**Table 1:** Effect of breed, feed and packaging method on the CIELab colour parameters of sheep *Semimembranosus* and *Vastus lateralis* after 10-day retail display.

Effect	Treatment	L* (Lightness)		a* (Redness)		b* (Yellowness)		h* (Hue Angle)		C* (Chroma)	
		Coeff	*p*-Value	Coeff	*p*-Value	Coeff	*p*-Value	Coeff	*p*-Value	Coeff	*p*-Value
	*Semimembranosus* Constant ^1^	37.5 ± 1.37		20.41 ± 1.02		19.63 ± 0.98		43.67 ± 2.33		28.55 ± 1.16	
Breed	Merino	−2.4 ± 2.56	<0.001	1.87 ± 1.96	0.383	−3.6 ± 1.93	0.278	−8.35 ± 4.63	0.402	−1.14 ± 2.28	0.927
Feed	SCF ^2^	2.7 ± 2.44	0.194	−1.89 ± 1.82	0.418	−1.70 ± 1.78	0.712	1.86 ± 4.27	0.671	−2.93 ± 2.12	0.29
	SCM ^3^	−2.3 ± 2.44	0.194	0.46 ± 1.82	0.418	1.01 ± 1.78	0.712	2.14 ± 4.27	0.671	0.68 ± 2.12	0.29
Packaging	HioxMAP ^4^	9.91 ± 1.57	<0.001	−11.71 ± 1.48	<0.001	−6.46 ± 1.24	<0.001	13.3 ± 3.24	<0.001	−12.64 ± 1.63	<0.001
	TrigasMAP ^5^	9.72 ± 1.57	<0.001	−11.39 ± 1.48	<0.001	−7.66 ± 1.24	<0.001	9.6 ± 3.24	<0.001	−13.44 ± 1.63	<0.001
	*Vastus lateralis* Constant ^6^	38.99 ± 1.52		19.33 ± 1.05		18.37 ± 0.84		42.96 ± 2.5		26.74 ± 1.07	
Breed	Merino	0.62 ± 2.48	<0.001	−2.94 ± 1.74	0.005	−5.18 ± 1.37	0.043	−2.75 ± 4.11	0.003	−5.33 ± 1.75	0.08
Feed	SCF ^2^	1.38 ± 2.49	0.63	−0.89 ± 1.72	0.104	−0.22 ± 1.39	0.174	1.52 ± 4.05	0.452	−0.78 ± 1.76	0.053
	SCM ^3^	1.58 ± 2.49	0.63	−0.35 ± 1.72	0.104	2.07 ± 1.39	0.174	4.19 ± 4.05	0.452	1.21 ± 1.76	0.053
Packaging	HioxMAP ^4^	9.74 ± 1.48	<0.001	−6.88 ± 0.98	<0.001	−5.99 ± 0.90	<0.001	2.9 ± 2.14	<0.001	−9.1 ± 1.09	<0.001
	TrigasMAP ^5^	10.45 ± 1.48	<0.001	−9.25 ± 0.98	<0.001	−6.59 ± 0.90	<0.001	6.72 ± 2.14	<0.001	−11.19 ± 1.09	<0.001

Coefficients ± standard error of differences (Coeff ± SED) and level of significance (*p*-values) are presented. ^1^ For *Semimembranosus* from a Composite lamb, finished on a standard pelleted diet containing grain and cereal hay, and retail displayed in vacuum skin packaging for 10 days. ^2^ SCF = standard pelleted diet containing 15% camelina forage hay. ^3^ SCM = standard pelleted diet containing 8% camelina meal (SCM). ^4^ HioxMAP = high-oxygen modified atmosphere packaging with 80% O_2_ and 20% CO_2_; ^5^ TrigasMAP = trigas modified atmosphere packaging with 50% O_2_, 30% N_2_ and 20% CO_2_; ^6^ For *Vastus lateralis* from a Composite sheep, fed with standard pelleted diet containing grain and cereal hay, and packaged in vacuum skin packaging.

**Table 2:** Effect of breed, feed and packaging method on water holding capacity and texture measurements of sheep *Semimembranosus* (topside) and *Vastus lateralis* (knuckle).

Effect	Treatment	Purge Loss (%)		Cooking Loss (%)		WBSF (N)		Hardness (N)		Cohesiveness		Chewiness (N)	
		Coeff	*p*-Value	Coeff	*p*-Value	Coeff	*p*-Value	Coeff	*p*-Value	Coeff	*p*-Value	Coeff	*p*-Value
	*Semimembranosus* Constant ^1^	6.3 ± 0.5		35.3 ± 0.9		25.3 ± 2.7		33.6 ± 1.5		0.16 ± 0.01		1.78 ± 0.32	
Breed	Merino	−0.6 ± 1	0.506	−2.3 ± 1.7	0.038	−0.4 ± 4.9	0.703	2.8 ± 4.3	0.001	−0.01 ± 0.03	<0.001	0.16 ± 0.8	<0.001
Feed	SCF ^2^	−0.3 ± 0.8	0.984	0.5 ± 1.3	0.566	3.2 ± 4.1	0.18	1.7 ± 2.3	0.952	−0.004 ± 0.02	0.334	0.21 ± 0.49	0.508
	SCM ^3^	0.5 ± 0.8	0.984	0.9 ± 1.3	0.566	4.6 ± 4.1	0.18	0.8 ± 2.3	0.952	0.02 ± 0.02	0.334	0.37 ± 0.49	0.508
Packaging	HioxMAP ^4^	0.4 ± 0.6	0.014	−3 ± 1.1	<0.001	3.8 ± 3.1	0.001	3.0 ± 3.3	0.07	0.05 ± 0.02	<0.001	1.14 ± 0.63	<0.001
	TrigasMAP ^5^	−0.5 ± 0.6	0.014	−3.3 ± 1.1	<0.001	5.1 ± 3.1	0.001	6.6 ± 3.3	0.07	0.03 ± 0.02	<0.001	1.07 ± 0.63	<0.001
	*Vastus lateralis* Constant ^6^	6.0 ± 0.5		35.3 ± 0.9		20.9 ± 1.2		32.9 ± 2		0.18 ± 0.02		2.04 ± 0.56	
Breed	Merino	−0.8 ± 1	0.101	−1.4 ± 1.6	0.348	0.3 ± 2.5	0.688	−4.8 ± 4.5	0.756	−0.01 ± 0.05	0.005	−0.08 ± 1.22	0.01
Feed	SCF ^2^	0.6 ± 0.8	0.936	1.3 ± 1.3	0.742	2.9 ± 1.9	0.117	−0.9 ± 2.9	0.034	−0.02 ± 0.03	0.926	−0.31 ± 0.85	0.551
	SCM ^3^	0.2 ± 0.8	0.936	1.1 ± 1.3	0.742	1 ± 1.9	0.117	−1.2 ± 2.9	0.034	−0.02 ± 0.03	0.926	−0.23 ± 0.85	0.551
Packaging	HioxMAP ^4^	−0.1 ± 0.7	0.031	−2.7 ± 0.9	<0.001	2.1 ± 1.7	0.763	0.3 ± 3.2	0.695	0.05 ± 0.03	<0.001	1.36 ± 0.82	<0.001
	TrigasMAP ^5^	0.3 ± 0.7	0.031	−4.9 ± 0.9	<0.001	1.4 ± 1.7	0.763	0.7 ± 3.2	0.695	0.04 ± 0.03	<0.001	1.11 ± 0.82	<0.001

Coefficients ± standard error of differences (Coeff ± SED) and level of significance (*p*-values) are presented. ^1^ For *Semimembranosus* from a Composite lamb, finished on a standard pelleted diet containing grain and cereal hay, and retail displayed in vacuum skin packaging for 10 days. ^2^ SCF = standard pelleted diet containing 15% camelina forage hay. ^3^ SCM = standard pelleted diet containing 8% camelina meal (SCM). ^4^ HioxMAP = high-oxygen modified atmosphere packaging with 80% O_2_ and 20% CO_2_; ^5^ TrigasMAP = trigas modified atmosphere packaging with 50% O_2_, 30% N_2_ and 20% CO_2_; ^6^ For *Vastus lateralis* from a Composite sheep, fed with standard pelleted diet containing grain and cereal hay, and packaged in vacuum skin packaging.

**Table 3:** Effect of breed, feed and packaging method on lipid and protein oxidation measurements of sheep *Semimembranosus* (topside) and *Vastus lateralis* (knuckle).

Effect	Treatment	TBARS (mg MDA·kg^−1^ Meat)		Total Carbonyl (nmol·mg^−1^ Protein)		Free Thiol Content (nmol·mg^−1^ Protein)	
		Coeff	*p*-Value	Coeff	*p*-Value	Coeff	*p*-Value
	*Semimembranosus* Constant ^1^	0.51 ± 0.33		1.52 ± 0.58		53.48 ± 3.14	
Breed	Merino	−0.13 ± 0.46	0.323	−0.11 ± 0.77	0.781	−14.87 ± 6.85	0.661
Feed	SCF ^2^	−0.4 ± 0.47	0.034	−0.66 ± 0.81	0.395	0.85 ± 5.02	0.37
	SCM ^3^	−0.28 ± 0.47	0.034	−0.50 ± 0.81	0.395	0.02 ± 5.02	0.37
Packaging	HioxMAP ^4^	4.05 ± 0.45	<0.001	1.75 ± 0.58	<0.001	−10.62 ± 2.52	<0.001
	TrigasMAP ^5^	1.80 ± 0.45	<0.001	1.74 ± 0.58	<0.001	−8.92 ± 2.52	<0.001
	*Vastus lateralis* Constant ^6^	0.29 ± 0.36		1.2 ± 0.29		54.62 ± 3.15	
Breed	Merino	0.14 ± 0.50	0.716	0.28 ± 0.41	0.404	−13.21 ± 6.51	0.424
Feed	SCF ^2^	−0.23 ± 0.54	0.06	−0.36 ± 0.42	0.162	0.36 ± 5	0.382
	SCM ^3^	−0.11 ± 0.54	0.06	−0.23 ± 0.41	0.162	−1.62 ± 5	0.382
Packaging	HioxMAP ^4^	2.59 ± 0.41	<0.001	2.06 ± 0.39	<0.001	−10.29 ± 2.43	<0.001
	TrigasMAP ^5^	2.67 ± 0.41	<0.001	1.96 ± 0.39	<0.001	−8.22 ± 2.43	<0.001

Coefficients ± standard error of differences (Coeff ± SED) and level of significance (*p*-values) are presented. ^1^ For *Semimembranosus* from a Composite lamb, finished on a standard pelleted diet containing grain and cereal hay, and retail displayed in vacuum skin packaging for 10 days. ^2^ SCF = standard pelleted diet containing 15% camelina forage hay. ^3^ SCM = standard pelleted diet containing 8% camelina meal (SCM). ^4^ HioxMAP = high-oxygen modified atmosphere packaging with 80% O_2_ and 20% CO_2_; ^5^ TrigasMAP = trigas modified atmosphere packaging with 50% O_2_, 30% N_2_ and 20% CO_2_; ^6^ For *Vastus lateralis* from a Composite sheep, fed with standard pelleted diet containing grain and cereal hay, and packaged in vacuum skin packaging.

## Data Availability

The data presented in this study are available on reasonable request.
